# Supplementary material for: Catechin versus MoS2 Nanoflakes Functionalized with Catechin: Improving the Sperm Fertilizing Ability—An In Vitro Study in a Swine Model
Source: Int J Mol Sci. 2023 Mar 1;24(5):4788. doi: 10.3390/ijms24054788 (PMC10003105; doi:10.3390/ijms24054788)
Supplement: Supplementary file 1 [file ijms-24-04788-s001.zip › ijms-2185666-supplementary.pdf]

Supplementary Materials S1

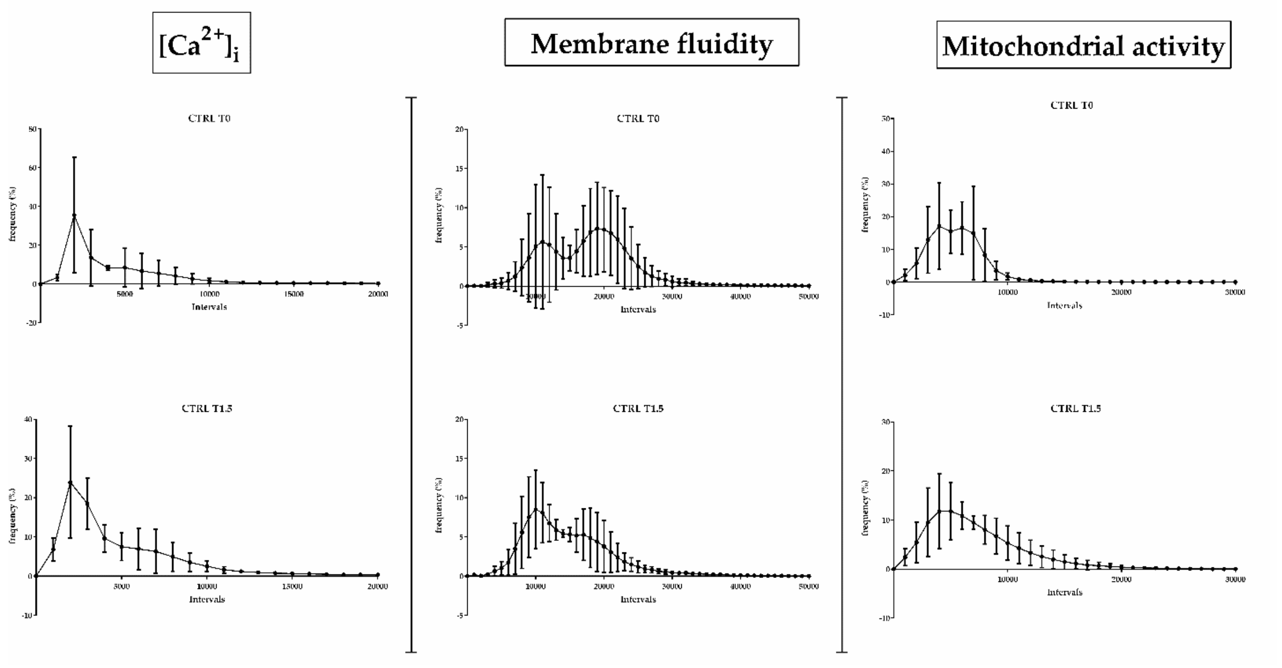

Supplementary Materials S1. CTRL group flow cytometry analysis after 0, 1.5 of capacitation.

Supplementary Materials S2

All full-length membranes and immunoblotting images from three independent experiments.

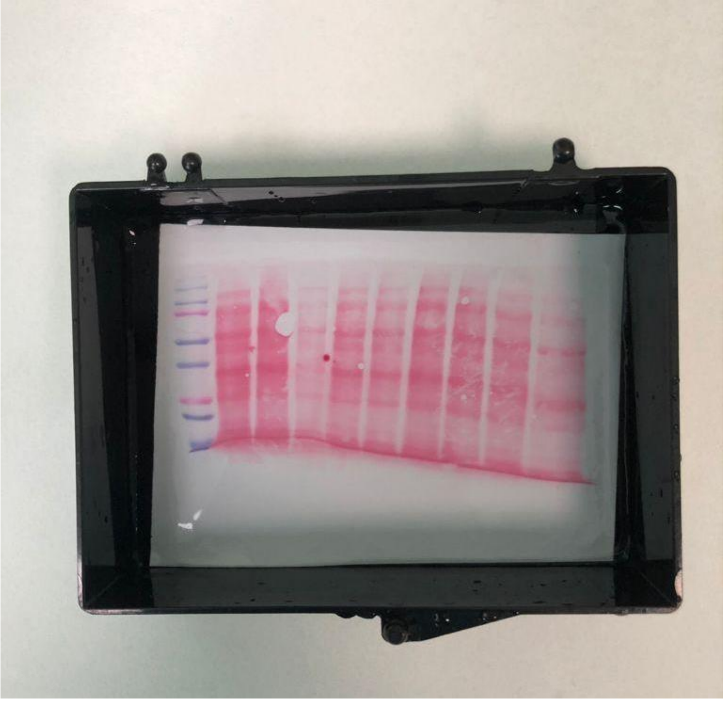

Supplementary Figure S1. Representative image of a membrane prior to hybridization. Blots used were mini size. This figure shows a membrane after Ponceau staining and prior to the incubation with the corresponding antibody

left **middle** **right**

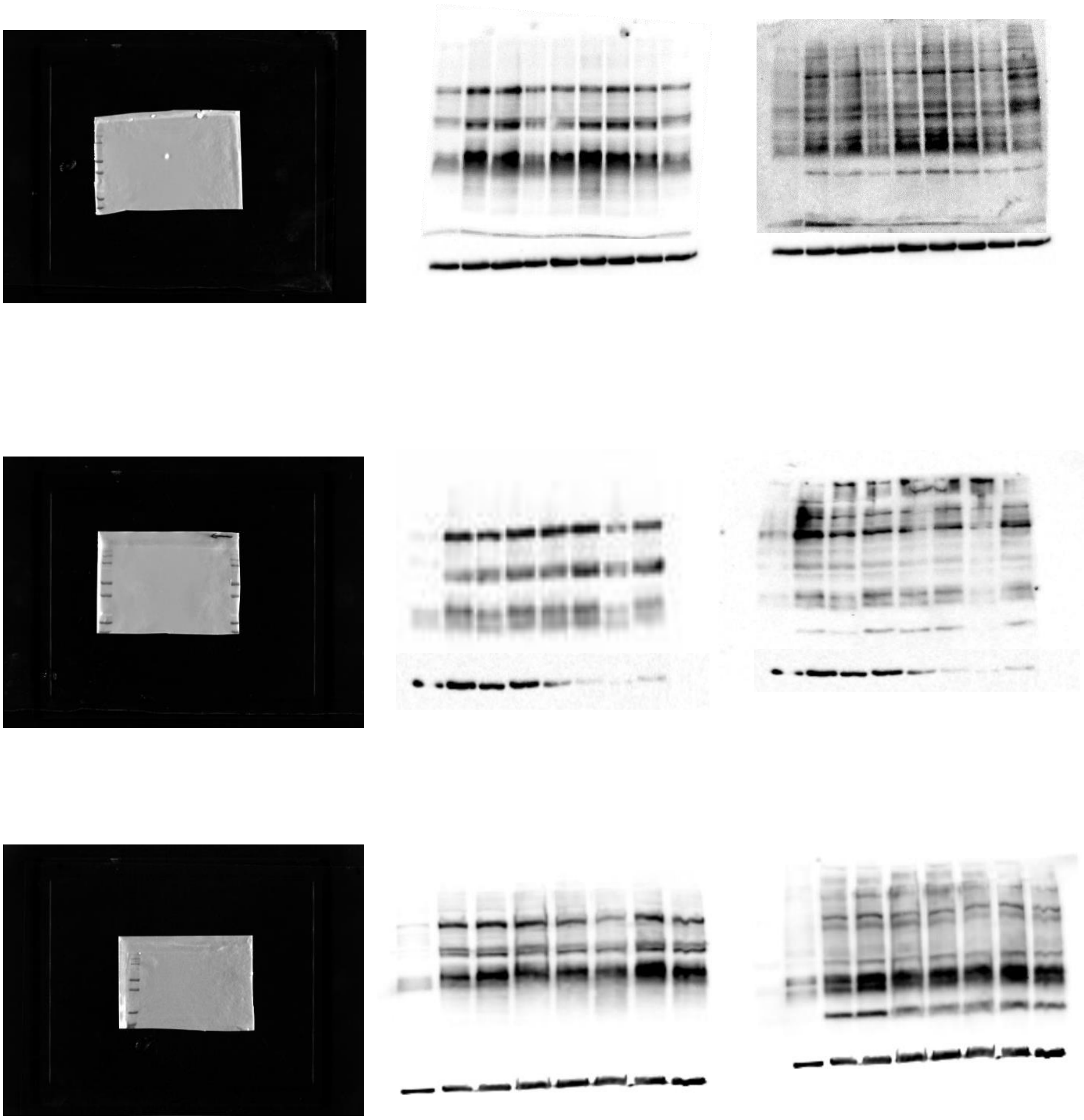

Supplementary Figure S2. Three representative blots with full-length membranes and membrane edges for anti-phospho-pKa antibody (Cell Signaling, Leiden, The Netherlands) and anti-phosphotyrosine antibody (Merck Millipore, USA). Left: molecular weight; middle: PKA activity and right: tyrosine phosphorylation patterns.
